# Thanatophoric Dysplasia With Concurrent Hydroureteronephrosis: A Rare Case Report From Rural Southern India

**DOI:** 10.7759/cureus.70842

**Published:** 2024-10-04

**Authors:** Pratheep V, Prakash Gambhir, Soundharya V

**Affiliations:** 1 Pathology, LifeCell International Private Limited, Chennai, IND; 2 Pathology, Sri Ramachandra Institute of Higher Education and Research, Chennai, IND; 3 Paediatrics, LifeCell International Private Limited, Chennai, IND; 4 Transfusion Medicine, Saveetha Medical College and Hospitals, Saveetha Institute of Medical and Technical Sciences, Saveetha University, Chennai, IND

**Keywords:** dwarfism, dysmorphic, hydronephrosis, rhizomelia, thanatophoric dysplasia

## Abstract

Thanatophoric dysplasia (TD) is a severe and typically fatal skeletal disorder caused by mutations in the FGFR3 gene, often leading to perinatal death. It is characterized by extreme short-limb dwarfism and, occasionally, associated anomalies such as hydronephrosis. Prenatal diagnosis, usually made in the third trimester through ultrasound and genetic testing, is crucial for guiding management decisions. Here, we report a case of TD with hydronephrosis diagnosed at 24 weeks of gestation in a 24-year-old primigravida from rural southern India. Ultrasound findings included significantly short and curved long bones, leading to the termination of the pregnancy. A post-termination examination confirmed the presence of dysplastic bones, a large head, and hydronephrosis, with histopathological analysis revealing obstructive uropathy. This case underscores the rarity of TD with hydronephrosis and highlights the importance of early and accurate prenatal diagnosis through ultrasound and molecular testing. Despite the challenges in diagnosing TD, especially when additional anomalies are present, early detection in the second trimester can play a crucial role in guiding genetic counseling and management decisions.

## Introduction

Thanatophoric dysplasia (TD) is a severe skeletal disorder marked by extreme short-limb dwarfism, which is typically fatal in the perinatal period. The incidence of TD is estimated to range from 1 in 20,000 to 50,000 live births [1]. This condition is caused by mutations in the *FGFR3 gene*, located on chromosome 4p16.3, which spans approximately 16.5 kb and comprises 19 exons [2]. The FGFR3 gene plays a critical role in regulating bone growth, and mutations result in abnormal skeletal development, leading to the characteristic features of TD [3].

TD is generally inherited in an autosomal dominant manner, with a high frequency of new mutations. However, rare instances of familial sporadic occurrence have been reported, suggesting the possibility of autosomal recessive inheritance in certain cases [4]. Most fetuses with TD do not survive postnatally, typically resulting in intrauterine demise or neonatal death within the first 48 hours of life. Nonetheless, there are cases where individuals with TD survive beyond infancy, although they usually have a significantly shortened lifespan, with death often occurring before the first decade of life [3,4]. Here, we present a case of TD associated with hydronephrosis, diagnosed in the second trimester in a rural area of southern India.

## Case presentation

A 24-year-old primigravida at 24 weeks of gestation presented for routine prenatal care. She was euglycemic, normotensive, Rh-positive, and non-immunocompromised. She had a history of a non-consanguineous marriage and no family history of genetic defects. Additionally, her trisomy screening results from non-invasive prenatal testing (NIPT) indicated a low risk. An ultrasound scan (USG) at 24 weeks revealed a single intrauterine pregnancy, but the fetus exhibited significantly shortened and curved long bones, suggestive of rhizomelia and mesomelia. Based on these findings, the decision was made to terminate the pregnancy.

Fetogram findings

**Figure 1:**
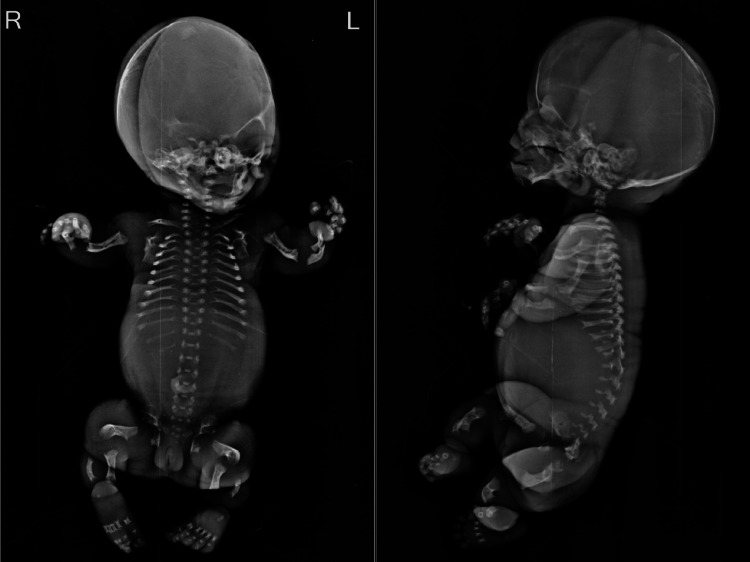
Fetogram showing anteroposterior and lateral views with severely dysplastic bones, including a large cranium, shortened limb bones with bowing, short ribs, and platyspondyly.

The fetogram (Figure 1) revealed severely dysplastic bones, including bowing of both the proximal and distal bones of the limbs, metaphyseal flaring of long bones, flattening of the vertebral bodies, a narrow chest with short ribs and anterior cupping, small iliac bones, reduced size of the pubic and ischial bones, and a poorly ossified large skull.

Physical examination

**Figure 2:**
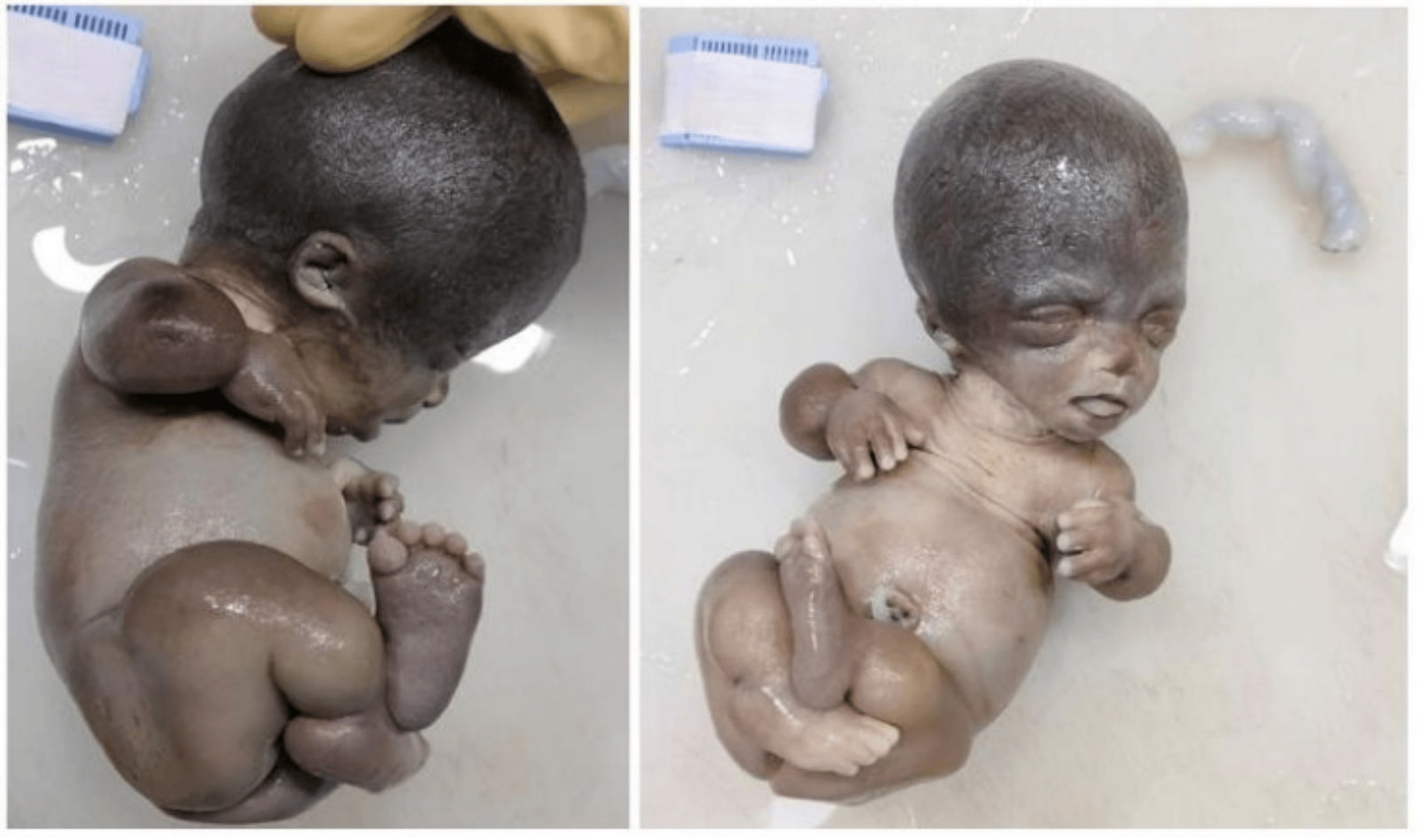
Gross images showing a hypercoiled umbilical cord, a large head with dysmorphic facial features, and generalized limb shortening.

**Figure 3:**
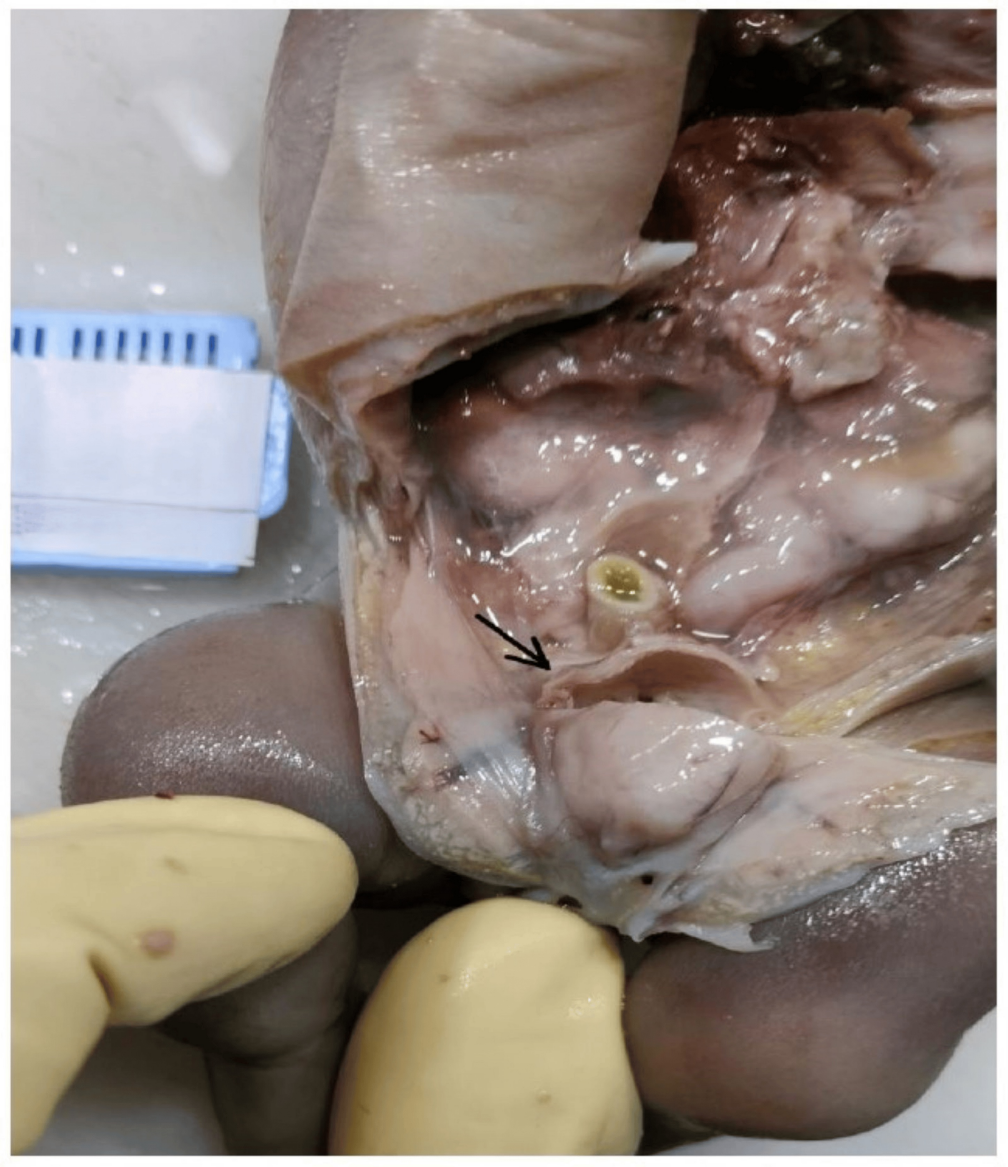
The arrow highlights a cut-open, enlarged, and dilated bladder.

**Figure 4:**
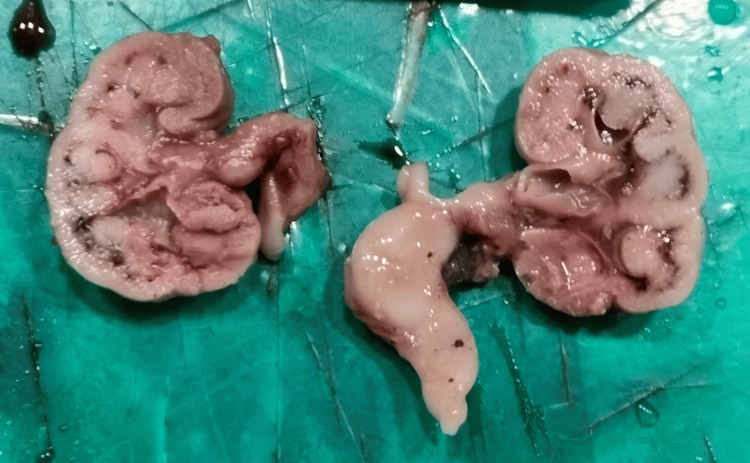
Cut surface of kidneys showing a dilated pelvicalyceal system and ureter.

Post-termination examination of the fetus (Figure 2) revealed a large head with hypertrichosis, dysmorphic facial features, and a focally hypercoiled umbilical cord. The fetus exhibited shortening of both upper and lower limbs, an enlarged abdomen, a narrow thorax, and an enlarged bladder (Figure 3) with dilated ureters and bilateral renal pelviectasis (Figure 4).

Histopathological examination

**Figure 5:**
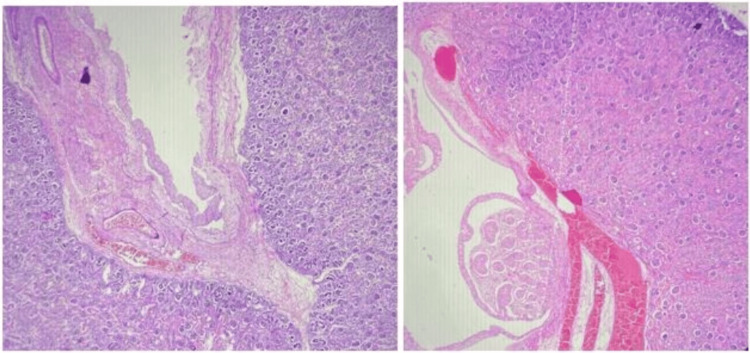
Microscopic image showing the dilated pelvicalyceal system of the kidneys at 200x magnification.

Microscopic examination (Figure 5) of the kidneys showed a dilated pelvicalyceal system with focal blunting of the calyces and thinned-out bladder wall, consistent with hydronephrosis. These findings are likely secondary to the underlying dysplastic skeletal abnormalities.

## Discussion

The term 'thanatophoric' is derived from the Greek words 'thanatos,' meaning 'death,' and 'phoros,' meaning 'bearing.' Thanatophoric dwarfism was first described in 1967 as a specific form of chondrodystrophy characterized by extreme short-limb dwarfism and perinatal lethality [2]. In 1977, the condition was renamed thanatophoric dysplasia at the Second International Conference on the Nomenclature of Skeletal Dysplasias [5]. TD is classified into two clinical subtypes based on skull and femur morphology: Type I and Type II. Type I, the more common form, features short, bowed 'telephone receiver' femurs without a cloverleaf skull, while Type II is distinguished by straight femurs and a trilobal cloverleaf skull. Both types share common characteristics, including short ribs, a narrow bell-shaped thorax, relative macrocephaly, specific facial traits, short fingers and toes, hypotonia, and redundant skin folds on the limbs [6].

The sonographic features of TD, including a narrow thorax, protuberant abdomen, hydramnios, ventriculomegaly, cloverleaf skull, and marked shortening of major long bones, are crucial for prenatal diagnosis. However, in many cases, diagnosis is difficult without advanced imaging techniques such as 3D anatomy scans or molecular confirmation through genetic testing [7]. Diagnosis can be further confirmed with autopsy and histopathological examination [8]. Amniocentesis may be performed for molecular analysis to confirm the FGFR3 mutation [2].

TD is part of a spectrum of dysplasias associated with FGFR3 mutations, including achondroplasia and hypochondroplasia, which serve as differential diagnoses [9]. Achondroplasia is the most common non-lethal skeletal dysplasia, characterized by relatively consistent clinical and radiological features, while hypochondroplasia presents a milder phenotype with more variability [10,11]. Long-term survival in TD is rare, particularly in type II, but type I TD has seen some cases of survival beyond the perinatal period [12].

In this case, the presence of hydronephrosis added complexity to the clinical picture. Hydronephrosis in TD may result from obstructive uropathy due to abnormal skeletal development or be a secondary feature of the dysplastic process. The association of TD with hydronephrosis is rare and not well-documented, making this case particularly noteworthy.

## Conclusions

TD is usually diagnosed prenatally, with the third trimester being the most reliable time for detection. However, as illustrated by our case, TD can also be accurately diagnosed in the second trimester. While the use of abdominal CT may enhance diagnostic precision, it does not alter the poor prognosis associated with this condition. Early and accurate diagnosis through ultrasound, supported by molecular testing, is crucial in the second trimester, as it facilitates informed genetic counseling and consideration of pregnancy termination in cases of lethal anomalies. Given that most TD cases occur sporadically, it is essential to reassure families that the recurrence risk is low, typically affecting only one previous fetus, and that extended family members are not at increased risk.
